# A Theoretical Investigation into the Homo- and Hetero-leptic Cu(I) Phosphorescent Complexes Bearing 2,9-dimethyl-1,10-phenanthroline and bis [2-(diphenylphosphino)phenyl]ether Ligand

**DOI:** 10.3390/ma15207253

**Published:** 2022-10-17

**Authors:** Lu Shen, Yu-Yang Wang, Teng-Fei He, Lu-Yi Zou, Jing-Fu Guo, Ai-Min Ren

**Affiliations:** 1 Department of Science, Jilin Jianzhu University, Changchun 130118, China; 2 Laboratory of Theoretical and Computational Chemistry, Institute of Theoretical Chemistry, College of Chemistry, Jilin University, Changchun 130023, China; 3 School of Physics, Northeast Normal University, Changchun 130024, China

**Keywords:** homo-ligand, hetero-ligands, phosphorescent quantum efficiency, spin–orbit coupling

## Abstract

Cu(I) complexes have received widespread attention as a promising alternative to traditional noble-metal complexes. Herein, we systematically study the properties of Cu(I) complexes from homo- to hetero-ligands, and found the following: (1) hetero-ligands are beneficial to regulate phosphorescent efficiency; (2) when the hetero-ligands in a tetracoordinated Cu(I) complex are 1:1, the ligands coordinate along the d_x2-y2_ direction of Cu(I) ion, which can observably suppress structural deformation; (3) unlike the P^P ligand, the N^N ligand can enhance the participation of Cu(I) during the transition process; (4) the addition of an appropriate amount of P^P ligand can effectively raise the energy level of HOMO (highest occupied molecular orbital), enhance the proportion of LLCT (ligand–ligand charge transfer), and thereby increase the available singlet emission transition moments which can be borrowed, thus promoting the radiative decay process. As a result, this work provides a detailed understanding of the effects of different ligands in Cu(I) complexes, and provides a valuable reference and theoretical basis for regulating and designing the phosphorescent properties of Cu(I) complexes in the future.

## 1. Introduction

Transition-metal complexes, such as Ir [1], Pt [2], Os [3], Au [4] and other complexes are widely used in solar energy conversion [5], chemical sensing [6], photocatalysis [7] and displays, as well as biological molecular probes and materials in the science industry [8]. These complexes are especially attractive as candidates for efficient OLEDs, as they can both harvest singlet and triplet excitons and show higher phosphorescence quantum yields [9]. However, there are some potential drawbacks of transition-metal complexes, including high costs and environmental concerns. Cu(I) complexes are ideal candidates for metal complex luminescent materials because of their low cost, environmental friendliness, lack of MC (metal-centered excited state) transitions [10], and diverse coordination structures [11,12,13,14]. The coordination behavior depends on the electron structure of Cu(I). Because of the fulfilling of the d-orbital (d^10^ configuration), the ligand coordination sites around the metal center have the least electrostatic repulsion from each other and have tetrahedral structures due to the symmetrical distribution of their charges. It is clear that d^10^ configuration prevents the d–d centered metallic electron transition in Cu(I) compounds [10].

The luminescence performance of Cu(I) complexes have been extensively investigated. Initially, the four coordination Cu(I) complexes [Cu(N^N)^2^]^+^ and [Cu(P^P)^2^]^+^ were studied [15,16,17]. There have been many reports about homogeneous complexes in the form of [Cu(N^N)^2^]^+^ (N^N = phenanthroline). For example, the introduction of larger ligands at the 2 and 9 positions of [Cu(N^N)^2^]^+^ can inhibit the quenching of the complexes, the effective size increases in the following order: methyl< n-butyl≈n-octyl< neopentyl< sec-butyl [15]. For the past few years, the Cu(I) complex [Cu(N^N) (P^P)]^+^ (POP = bis [2-(11)(diphenylphosphino)phenyl]ether, N^N = 1,10-phenanthroline]) has attracted more attention [18,19]. One of the most important reasons is that N^N ligands (such as phenanthroline) are strong electron-acceptors due to their large π characteristics. It is regarded as the target of metal-to-ligand charge transfer, which affects the LUMOs (lowest unoccupied molecular orbital) of the complexes [20]. The other reason is that, as an electron-donor, the phosphine ligands can stabilize the HOMOs (highest occupied molecular orbital) and can also enhance the luminous quantum yield of Cu(I) complexes, so these complexes have a longer excited state life and color adjustability [21]. The luminescence performance of [Cu(N^N) (P^P)]^+^ complexes was improved by modifying the ligand species, such as POP or dppb (1,2-bis-(diphenylphosphino)-benzene) ligands [14,22]. These hybrid ligand complexes improve luminescence performance by reducing the flattening of the Cu(I) center, so as to obtain a lower non-radiative decay [23].

**Figure 1:**
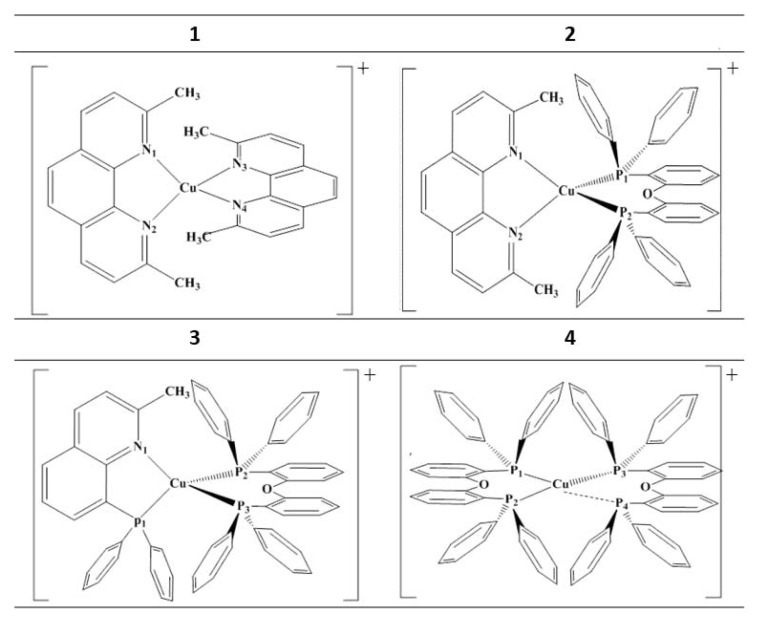
Chemical structures of the studied complexes.

Therefore, the study of hetero-leptic Cu(I) complexes, with an evolution from the N^N ligand to the P^P ligand, is a very critical step towards finding an alternative to the traditional precious metal complexes in electroluminescent devices. However, the current research in this area mainly focuses on the properties of delayed fluorescence and the mechanism of light emission [24]. Some scattered experimental and theoretical studies of nitrogen–phosphorus hetero-ligands have mainly focused on the properties and performance issues of nitrogen–phosphorus hetero-ligands in dinuclear or trinuclear complexes. There are relatively few studies devoted to how does the cooperation between different ligands effect the luminescence properties. Therefore, in this study, four Cu(I) complexes (shown in Figure 1) with various ligands (gradually change from N^N ligands to P^P ligands) are investigated, to gain a better understanding of the effects of different N^N and P^P homo- and hetero-ligands on the phosphorescent quantum efficiency. We expect that this work can contribute to a detailed understanding of the effects of N^N and P^P homogeneous and heterogeneous ligands in Cu(I) complexes on the electronic structure and light emission properties, to provide a reliable theoretical basis for regulating and rationally designing the luminescence properties of Cu(I) complexes.

## 2. Computation Methods

All the calculations in this paper are completed by Gaussian 09 package [25]. Density functional theory (DFT) [26] and the unrestricted DFT method were used to optimize the S_0_ state and T_1_ state geometry of the complexes. The vibration frequencies were calculated to make sure all the structures are stable and have no imaginary frequency. The LANL2DZ basis set was used for Cu(I) atom [27], and the 6–31G(d) basis set was employed for all nonmetallic atoms [28]. The transition properties of Cu(I) complexes were calculated using a polarizable continuum model [29] (CH_2_Cl_2_) by time-dependent density functional (TD–DFT) [30].

**Figure 2:**
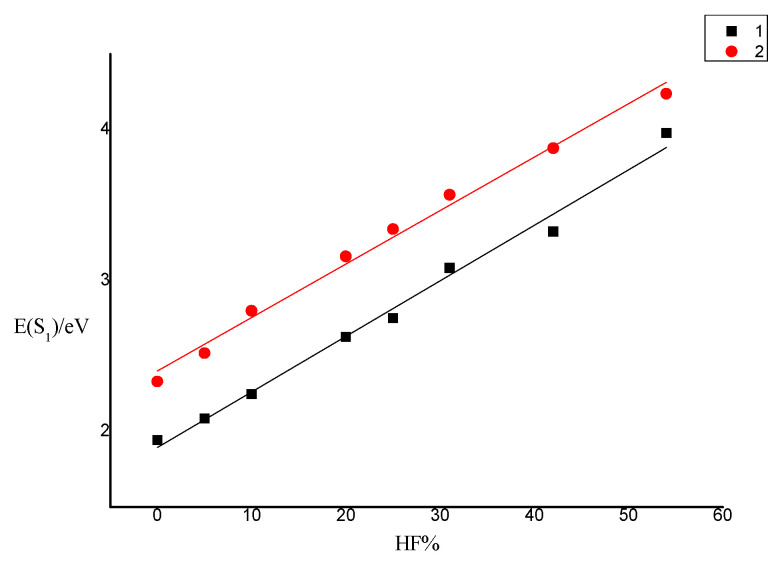
Dependence of *E*(S_1_) of complex **1** on the HF% in TD–DFT functionals.

It is well known that the optical properties of complexes are very sensitive to the selection of hybrid functionals. It has been reported that the calculation of the vertical excitation energy *E*(S_1_) by the TD–DFT method has an important dependence on the Hartree–Fock percentage (HF%) contained in functionals [31]. Taking complexes **1** and **2** as examples, functionals with different HF% were simulated: BLYP [32,33], MPWLYP1M [34], TPSSH [35], B3LYP [36], PBE0 [37,38], MPW1B95 [38], BMK [39], M06-2x [40], the vertical excitation energy *E*(S_1_) data in CH_2_Cl_2_ solvent is shown in Table 1. The fit curve of *E*(S_1_) as a function of HF% is shown in Figure 2. The results show that *E*(S_1_) is positively correlated with HF%. When HF% < 54%, a good linear relationship (R_1_^2^ = 0.99521, R_2_^2^ = 0.99588) between *E*(S_1_) and HF% is observed (Figure 2).

**1**: E_1_(S_1_) = 1.89465 + 0.03879HF% (1)

**2**: E_2_(S_1_) = 2.40138 + 0.03541HF% (2)

In the experiment, the *E*(S_1_) of complex **1** was equal to 2.71 eV, according to Equation (1), the best HF% is 21.02%. According to Equation (2), the best HF% is 23.60% with the value of experiment (*E*(S_1_) = 3.24 eV). Therefore, B3LYP and PBE0 are considered as a suitable functionals. Using B3LYP and PBE0 functionals to calculate the absorption spectrum and emission spectrum of complexes **1**–**3**, the calculated data are listed in Tables S1 and S2. The calculated results of PBE0 are very consistent with the experimental values, so PBE0 can be used to accurately describe the properties of this series of complexes.

## 3. Results and Discussion

### 3.1. Geometric and Electronic Structures

The structure diagrams of the studied Cu(I) complexes are shown in Figure 2. The important bond length and bond angle of the complexes are listed in Table S3. The isolated single complex that extracts from the single crystal structure is used as the initial structure of the optimization, so the effect of intermolecular interaction is lacking, the bond lengths of Cu–N and Cu–P in the optimized ground state geometry are evidently about 3–5% longer than those in the crystal structure. Compared with the experiment, the bond angle and dihedral angle have smaller errors (1–3%).

It can be seen from Table S3, in the S_0_ state, from the homo-coordination complexes composed of N^N ligands, to hetero-coordination complexes of N^N ligands and P^P ligands, and to homo-coordination complexes of P^P ligands (the complexes are from **1** to **4**), the bond length of Cu–N and Cu–P gradually increases. This means that the Cu(I) move away from the ligands (N^N ligands and P^P ligands) gradually due to the change of the ligand. This can weaken the coordination between the metal and the ligands, which is not conducive to the MLCT process. For complex **4**, the bond length of Cu–P_4_ is 4.1866 Å, which is far beyond the range of the bond length of a single bond (the van der Waals radius of Cu and P are 1.86 Å and 1.96 Å, respectively) [41]. It means that the metal center does not form a bond with the P_4_ atom. In addition, the bond angle of complex **4** is P_1_–Cu–P**_2_**_:_ 113.14°; P_2_–Cu–P**_3_**_:_ 120.05°; P_1_–Cu–P**_3_**_:_ 124.38°; the sum is 357.57°, which is very close to 360°. The geometric structure of the three coordinated P atoms is a plane-like triangle instead of a tetrahedron. The reason why the fourth P atom does not bond with Cu(I) is probably because of the steric hindrance of the diphenyl ether skeleton and POP ligand [42].

Comparing the structural changes from the S_0_ to the T_1_ state of each complex, we found that the bond length of Cu–N gradually decreases, while the Cu–P bond length becomes longer, that is, Cu(I) is closer to the N^N ligand and away from the P^P ligand during the change from the S_0_ state to T_1_ state. It is found that the change in DHA1 (the dihedral angle between the ligands) has been suppressed by the addition of the P^P ligand (**1**: 89.64°→60.56°, **2**: 82.36°→68.52°, **3**: 83.35°→83.44°, **4**: 82.33°→80.01°), which may bring a smaller structure distortion between the S_0_ and T_1_ states. Pymol software was used to superimpose the molecular structure of two states and calculate the RMSD (root mean square deviation) [43]. The data are shown in Figure S1. The results indicate that the structural distortions of complexes **1**, **2**, **4** decrease with the addition of the P^P ligand. Although the DHA1 of complex **3** is closest to 90° and the change from the S_0_ to T_1_ state is the smallest (Δ = 0.09°) one, complex **3** has the largest RMSD. This can be attributed to the asymmetric coordination of N^P ligand, which lead to an imbalance on the donating and accepting electrons, then result in a large distortion between the S_0_ and T_1_ state.

### 3.2. Natural Population Analysis

In order to deeply investigate the relationship between the diverse ligands, the Natural Population Analysis is applied. The data are listed in Table 2. During the change of the complexes from **1** to **4**, the net charge of Cu(I) ion also decreases and then increases (**1**: 0.5615e, **2**: 0.3633e, 3:0.2257e, **4**: 0.5070e). These mean that the Cu(I) ion tends to lose charge from the homo-coordination (complexes **1** and **4**) to the hetero-coordination (complexes **2** and **3**). It is also found that the tends of lose charge decreases with the increasing of the number of P atoms.

As shown in Table S4, the net charges of the N atoms are negative, while the net charge of P atoms are positive. This means that the net charge of the central Cu(I), N and P atoms can be strongly affected by the change of the ligands. The charge distributions of the two ligands are similar due to having a homo-coordination.

From the S_0_ state to the T_1_ state, the change of net charge of Cu(I) ion in complex **1** is smaller than that of other complexes (**1**: Δ = 0.0002e). According to the result, complex **3** may have the highest metal participation in the charge transition process, while the complexes with hetero-coordination (complexes **2** and **3**) may have stronger charge transitions between the two ligands (LLCT) and between the intra-ligands (ILCT).

### 3.3. Ionization Energy (IP) and Electron Affinity (EA) and Reorganization Energy

It is critical to study whether the redox potential of the material complexes in a multilayer device can constitute a suitable barrier gradient, and thus allow different carriers to pass through smoothly. For the material of the OLED emission layer, low injection energy barrier and balanced charge transport ability are the goals pursued by design materials. Ionization energy (IP) and electron affinity (EA) are used to assess the ability of material complexes to lose and accept an electron. We calculated the complex’s IP and EA using PBE0/6-31G (d), and the results are listed in Table 3. As the addition of the P^P ligand, the IP and EA values of the complex gradually decrease, which means that the hole injection barrier of the complex is gradually reduced, and the electron injection barrier is heightened. That is the possibility of becoming a hole transport material increase.

The reorganization energy (*λ*) represents the geometrical relaxation energy experienced by the material complexes when they are injected and transferred a hole/electron. According to the Marcus formula, the reorganization energy is located in the exponential term of transfer rate formula, so it is one of the important factors that limit the size of the transmission rate. From Table 3, *λ*_electron_ of all complexes are smaller than λ_hole_, indicating that the electron transport rate of these complexes will be faster than the hole transport rate. The main reason is that the energy required to extract holes (HEP value) is much higher than the electron extraction energy (EEP value), that is, the holes have deeper traps. Compared with the hetero-leptic complexes **2** and **3**, the electron-reorganization energies of the homo-leptic compounds 1 and 4 are both smaller, while the hole reorganization energy values are larger. The electron and hole reorganization energy values of complex **2** are closer to each other (the difference between them: **1**: 0.51, **2**: 0.15, **3**: 0.27, **4**: 0.75), so complex **2** as OLED light-emitting layer material will exhibit better electron/hole transport balanced performance and higher luminescent efficiency. Compared with the excellent electron transport material Alq3 the EA value of Alq3 is 3.0 eV) [44], it can be seen that the EA values of the complexes **1**, **2**, **3** are close to it or larger than it, indicating as an OLED material they have a more suitable electron affinity potential.

### 3.4. Frontier Molecular Orbitals

**Figure 3:**
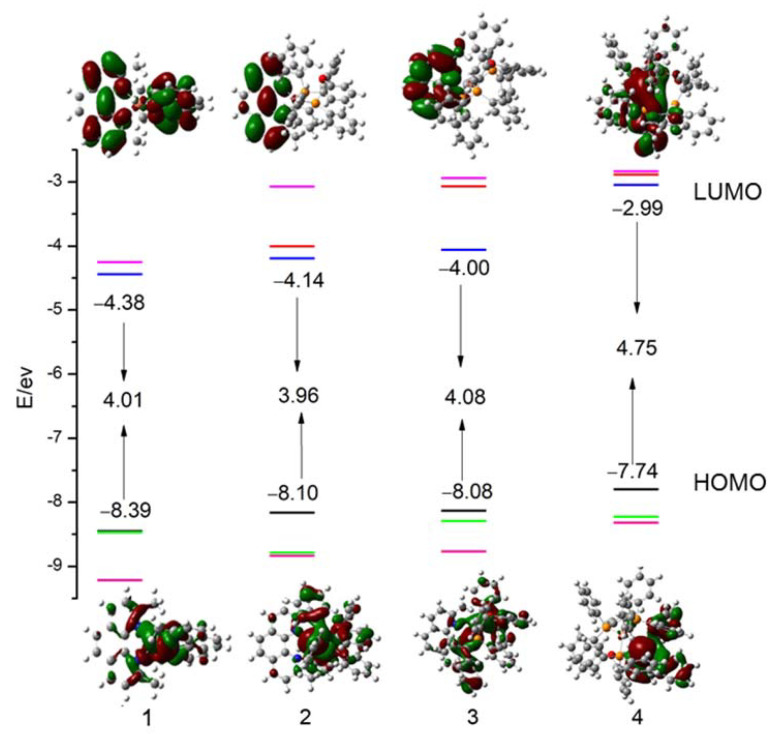
Frontier molecular orbital energy levels, the energy gaps ∆*E*_H-L_ and the electronic density contours of the frontier molecular orbital in the S^0^ state for studied complexes.

It is important to analyze the highest occupied molecular orbital (HOMO) and lowest unoccupied molecular orbital (LUMO) of the complexes. In order to more intuitively observe the effect of structural changes on the electronic properties, the energy levels of HOMOs, LUMOs, and HOMO-LUMO energy gaps (∆*E*_H-L_) were obtained by using the DFT method as shown in Figure 3.

Firstly, the electron cloud distribution of Cu(I) should be taken into consideration. For complex **1**, which has two homo-coordination N^N ligands, Cu(I) has the highest proportion (70.5%) in the HOMOs, and the proportion of two N^N ligands are same (14.8%). Because of the existence of the non-bonding P_4_ atom, complex **4** has a different proportion of the P^P ligands, and the lowest proportion of Cu(I) in the HOMO. When Cu(I) ion is bonded to the hetero-coordination ligands (complexes **2** and **3**), the proportion of Cu(I) in the HOMO decreases by about 30% compared to complex **1**, and the HOMO is mainly localized on the P^P ligands.

Secondly, the changes in the molecular orbital in the two ligands are discussed. It is found that with the addition of the P^P ligand, the electron cloud distribution of the HOMO changes significantly. The electron cloud moves from the N^N ligand to the P^P ligand gradually (the proportion of P^P ligand in the HOMO: **1**: 0%, **2**: 57.5%, **3**: 64.3%, **4**: 100%). However, the LUMOs mainly locate on the N^N ligands (the proportion of N^N ligand in the LUMO: **1**: 96.6%, **2**: 92.3%, **3**: 98.5%%, **4**: 0%). For complex **3**, it is clear that the LUMOs mainly locate on the quinoline group in N^P ligand. The results indicate that P^P ligands have stronger electron donor properties than N^N ligands among complexes with hetero-coordination. However, in the complexes with homo-coordination, the N^N ligands are both the electron-donors and electron-acceptors. As a result, the advantage of N^N ligand as an electron-acceptor is not significant, which may not be beneficial to the charge transition. From the above discussions, we know that the location of Frontier molecular orbitals (MOs) changed with the addition of P^P ligands, which led to a significant variation of the energy levels of HOMOs and LUMOs. The energy level of the HOMOs and LUMOs rise observably with the addition of the P^P ligands. In the meantime, the energy gaps display little change (**1**: Δ*E*_H-L_ = 4.01 eV, **2**: Δ*E*_H-L_ = 3.96 eV, **3**: Δ*E*_H-L_ = 4.08 eV). For complex **4**, the energy of the LUMO rises to 2.99 eV due to the number of P^P ligands changed from **3** to **4**. This means that the energy of the LUMO is increased by the electron cloud distribution of the P^P ligands. This also indicates that the photodynamic stability increased with the addition of the P^P ligands. In addition, complex **4** has a strong reducibility, which can be attributed to the uncoordinated P atom and large steric hindrance.

### 3.5. Absorption and Emission Spectra in PCM Solvent

#### 3.5.1. Absorption Spectra

**Figure 4:**
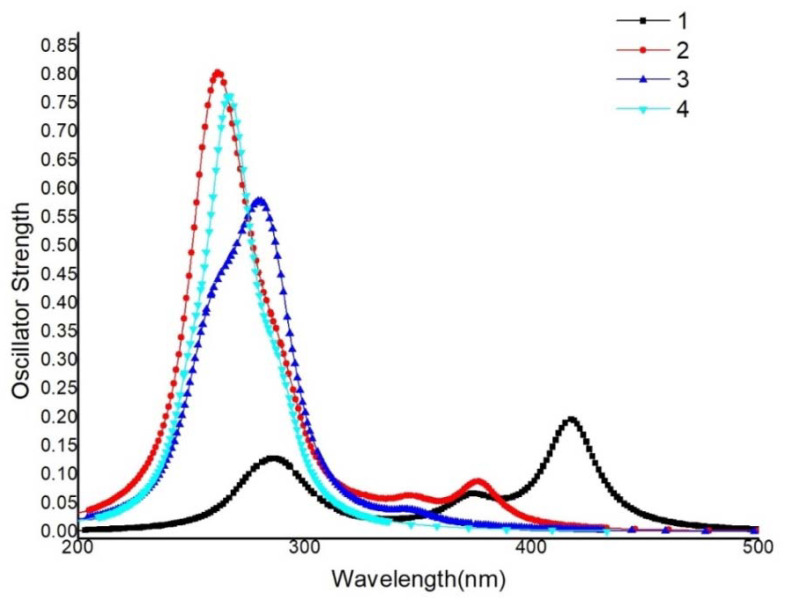
Simulated absorption spectrum of these complexes in PCM solution (CH_2_Cl_2_).

Based on the optimized structure of the S_0_ state and considering the solvent effect of CH_2_Cl_2_, the simulated absorption spectrums are shown in Figure 4. The calculated wavelength, oscillator strength, transition composition and the experimental values are listed in Table S6. From Table S6, it is clear that the calculated values agree well with the experiment (**2**: λ_max_^abs^=376.5 nm, λ^expt^=383 nm, **3**: λ_max_^abs^ =347.2 nm, λ^expt^ =350 nm,) [45], which indicates that the PBE0 functional can simulate the electronic properties well. Notably, compared to complex **3**, complex **2** has the larger oscillator strength, which may be conducive to the improvement of phosphorescence efficiency. 

For the absorption beyond 300nm, the maximum absorption peak of complex **1** is at 417.2 nm, corresponding to the mixed transition of H−1→L+1 (56.2%) and H→L (44.2%), which is regarded as ML_N_L_N_CT from the metal (Cu, d_xz_, d_xy_) to the two ligands. There are no other absorption peaks for other three complexes in this region, indicating that the addition of the P^P ligands will blue-shift the absorption spectrum. Complex **2** has a relatively weak absorption peak at 376.5 nm, which is mainly derived from the transition of H→L (92.5%). That is a mixture of metal (Cu, d_x2-y2_) to ligand (N^N) charge transfer (ML_N_CT) and P^P ligand to N^N ligand charge transfer (L_P_L_N_CT). Complex **3** has a weak absorption at 347.2 nm, corresponding to H−1→L (87.1%) and H→L (10.6%), which is also a mixture of ML_N_CT and L_P_L_N_CT, but Cu(d_xy_, d_x2-y2_) participates in charge transfer. There is no absorption peak in the long wavelength region for complex **4**. The above facts indicate that the addition of the P^P ligand causes a blue shift in the absorption spectrum.

**Figure 5:**
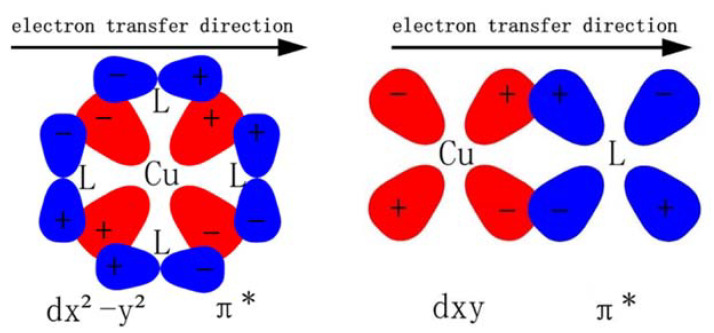
A schematic illustration of the symmetry of the ligand’s π-orbital with the d-orbital of Cu(I) and the charge transfer of the formed metal→ligand.

In short, in complex **2**, the N^N ligand coordinates with Cu(I) along the orbital direction of d_x2-y2_, then form a charge transfer transition from metal (Cu, d_x2-y2_) to ligand (N^N, π*). The other complexes, such as the homo-coordinated complex **1** and **4**, the hetero-coordinated complex **3**, all coordinate along the orbital direction of (d_xz_, d_xy_) and d_yz_, and therefore have a similar CT transition from metal (Cu, d_xz_/d_xy_/d_yz_) to the ligand (N^N and P^P, π*). This illustrates that different numbers of phosphorus atoms in ligands lead to different ligand fields with different metal d-orbitals (Figure 5), resulting in different degrees of structural deformation by way of the excited process, and finally affecting the luminescent efficiency. A detailed analysis of the effect on phosphorescent quantum efficiency will be further discussed below.

#### 3.5.2. Phosphorescent Spectrum

Based on the structure of the T_1_ state optimized by the UPBE0/6-31G (d) and LAN2DZ method, the emission spectra of all complexes in dichloromethane solution are listed in Table 4. It is found that the phosphorescent transitions of the four complexes mainly occurred from the HOMO to LUMO, so the properties of the transition can be replaced by the discussion of the HOMO and LUMO. The orbital compositions of the T_1_ state are listed in Table S7. Complex **1** has the highest ^3^MLCT ratio because of the highest proportion of Cu(I) in the HOMO. The ratios of complexes **2** and **3** are lower than complex **1.** Complex **4** has the lowest ^3^MLCT proportion owing to the lowest composition of Cu(I) in the HOMO (only 0.4%). Further analyzing the contribution of each fragment to the HOMOs and LUMOs, N^N ligands exhibit strong acceptor properties in the T_1_ state. When the P^P ligand is coordinated with Cu(I), the P^P ligands exhibit strong donor properties, that is, the contribution of the P^P ligand in the HOMO reaches 47.3%, and at the same time, the contribution of Cu(I) in the HOMO decreases to 39.4%. When the large π-conjugation in the N^N ligand is destroyed and replaced with a diphenylphosphine ligand (complex **3**), the contribution of the N^P fragment to the HOMO is significantly increased. When all the ligands are replaced by the P^P ligands, the proportion of Cu(I) in the HOMO reduces to 0.4% due to the strong donor properties, while the proportion of P^P (independent P) ligands reaches 98.3%. It should be pointed out that the LUMOs are not sensitive to the structure changes in P^P ligands, which is different from the HOMOs. The LUMOs of complexes **2** and **3** are mainly located on the N^N ligand. Based on the above discussion, the transition type of each complex can be identified as follows: the transition type of complex **1** is from the metal d-orbital to the π*-orbital of the N^N ligand (ML_N_L_N_CT). Complex **2** is a mixture of the transition from the π-orbital of the P^P ligand and the d-orbital of the metal to the π*-orbital of the N^N ligand (L_P_L_N_CT and ML_N_CT). Complex **3** is a mixture of the metal d-orbital and the π-orbital of the P^P ligand to the π*-orbital of the N^N ligand (ML_N_CT and L_P_L_N_CT). Complex **4** is from the π-orbital to the π*-orbital in the P^P ligand (independent P) (L_P1_L_P2_CT). From the above transition types, we found that complex **1** has only MLCT transition, so the participation of the N^N ligand is small. However, complex **4** has only ILCT transition in the P^P ligand, and the metal participation is close to zero. This may indicate that the phosphorescent radiative decay rate of complexes **1** and **4** may be unsatisfactory. However, for complexes **2** and **3**, they have a suitable proportion of MLCT and LLCT (between the N^N ligand and the P^P ligand) in the transition progress, which may be beneficial to the radiative decay.

In addition, complex **4** is easy to oxidize and is used as a catalyst for the organic reaction due to the non-bonding P atom. Therefore, complex **4** is not suitable to be a stable light-emitting material, and the experiments have confirmed that complex **4** did not emit light [46], which will be discussed in the following section.

### 3.6. The Quantum Yields ($\phi_{p}$)

The internal quantum yield for studied complexes is an important theoretical parameter to measure whether a complex can become a potential OLED material. The internal quantum yield ($\phi_{p}$) can be expressed as:

(3) $$\phi_{p}=\frac{k_{r}}{k_{r}+k_{nr}}$$

where *k*_r_ is the radiative decay rate and *k*_nr_ is the non-radiative decay rate.

#### 3.6.1. Radiative Decay

The radiative decay rate is derived from Einstein’s spontaneous emission coefficient and can be expressed as follows [47], the formula details are listed in the Supporting Materials:

(4) $$k_{r}^{\alpha }(T_{1}\to S_{0})=\frac{\eta^{3}E{\left(T_{1}\right)}^{3}}{1.5}{\left\{\sum_{n}\frac{\langle T_{1}^{\alpha }\left|{\hat{H}}_{SOC}\right|S_{n}\rangle }{E(S_{n})-E(T_{1})}\times {\left(\frac{f_{n}}{E(S_{n})}\right)}^{1/2}\right\}}^{2}$$

(5) $$k_{r,avg}^{RT}(T_{1}\to S_{0})=\frac{1}{3}\sum_{\alpha }k_{r}^{\alpha }$$

Based on Equation (4), the calculated radiative decay rates and experimental values are listed in Table 5. The related spin–orbit coupling (SOC) matrix elements and oscillator strength are listed in Tables S8–S11. It can be seen from Equation (4) that the radiative decay rate is directly related to the excited triplet energy *E*(T_1_), the excited singlet energy *E*(S_n_), the oscillator strength of S_n_→S_0_, the singlet–triplet energy difference (*E*(S_n_)−*E*(T_1_)) and the SOC matrix elements. The SOC matrix element is calculated by BDF Software [48,49].

From Table 5, it is found that complex **2** has the highest T_1_ state excited energy (*E*(T_1_) = 16,322 cm^−1^), which led the smallest energy difference between the singlets and triplets (*E* (S_n_)−*E* (T_1_)) (**1**: 3953 cm^−1^, **2**: 2121 cm^−1^, **3**: 4388 cm^−1^, **4**: 4483 cm^−1^). From Equation (4), we can see that a larger *E* (T_1_) and energy gap between *E* (S_n_) and *E* (T_1_) may bring a faster radiative decay rate. As a result, complex **2** may have a fast radiative decay rate. Furthermore, complex **1** has a smaller maximum SOC value than the other complexes (**1**: 83.61 cm^−1^, **2**: 212.51 cm^−1^, **3**: 202.96 cm^−1^, **4**: 169.26 cm^−1^), while the number of SOC matrix elements with the lager absolute value is also significantly less than other complexes. For complexes **2** and **3**, both the absolute value and the number of the SOC matrix elements are larger than complexes **1** and **4**, that is, complexes **2** and **3** have larger SOC, and complexes **1** and **4** have smaller ones. Concerning the calculation process, the radiative decay rate is not only related to the absolute value of SOC, but also closely to the cancelling of the positive and negative SOC values. Only when the singlet states have a large oscillator strength, effective SOC matrix element, and no-cancelling of the positive and negative value in the summation calculation, can the complexes have a large radiative decay rate.

Based on the data above, although the MLCT ratio in complex **1** is the highest, the SOC matrix element is weaker than the other complexes due to the single MLCT transition caused by the homogeneous N^N ligands. In the meantime, complex **1** has the lowest excited energy *E* (T_1_). Considering these two points, complex **1** has the lowest radiative decay rate. Complex **2** and complex **3** have similar MLCT ratios, and the mixed transition types (MLCT and LLC), and then have similarly sized SOC matrix elements. However, owing to the high matching of the oscillator strength and the SOC matrix element in complex **2**, the sum of the product for these two terms is significantly greater than that of complex **3** (see Supporting Materials). At the same time, complex **2** has the highest triplet energy due to the presence of pure P^P ligands. Finally, complex **2** has the fastest radiative decay rate. Because that one branch of the N^N ligand is replaced by the diphenylphosphine ligand in complex **3**, the triplet energy reduces (*E* (T_1_) = 13,780cm^−1^), and then its radiative decay rate becomes lower.

#### 3.6.2. Non-Radiative Decay

The non-radiative decay rate *k*_nr_ can be estimated by Equation (6) [50,51,52], in which the contributions of the low-frequency (*ω*_lf_ ≤ 1000 cm^−1^) and high-frequency (1700 > *ω*_hf_ > 1000 cm^−1^) vibration mode to *k*_nr_ are discussed, respectively. The related data are shown in Table 6. When the sum of the Huang–Rhys factor in low frequency is greater than 1 (*S*_lf_ > 1), the strong coupling limit is required. In case that the Huang–Rhys factor is less than 1 (*S* < 1) or ħω_M_ >> k_BT_, the weak coupling limit is required. Therefore, *k*_nr_ is estimated based on the strong coupling limit for *lf* modes and weak coupling limit for *hf* modes in the studied complexes. The details of the formula are listed in the Supporting Materials.

(6) knr(T1−S0)=2π〈T1|HSOC|Sm〉2ℏ2[2πℏ2(D12+P2)]−12exp[−(ΔE00−nMℏωM−λ1−μ)2πℏ2(D12+P2)]exp(−SMSMnMnM!)  

As shown in Table 6, the spin–orbit coupling (SOC) effect between the T_1_ and S_0_ states of complex **1** is significantly weaker than that of other complexes, with a value of only 0.014 cm^−1^. For phosphorescent complexes, besides the abundant aggregation of the triplet excitons, allowing the transition between the T_1_ and S_0_ state is necessary. That is, an appropriate spin–orbit coupling between the T_1_ and S_0_ state can successfully promote the transition of excitons gathered in the triplet states. Therefore, although complex **1** has a strong SOC effect between the T_1_ and S_1_ state, and a certain aggregation of the T_1_ state excitons, owing to the excessively small SOC effect between the T_1_ and S_0_ state, the phosphorescence emitting channel from the triplet state is blocked. This is consistent with the fact that complex **1** appears as a fluorescent emission in the experiment [15].

Reorganization energy is an important factor which can display the degree of electron–vibration coupling between two electronic states. Complex **1** has the lowest reorganization energy from the excited state to the ground state because of the high π-conjugation properties of the homogeneous N^N ligands. With the addition of the P^P ligand, large-sized phenyl-phosphine structures were introduced, and there is no obvious π-conjugated connection between the benzene rings comparing with the phenanthroline structure of **1**, resulting in a greater structural distortion between the S_0_ state and the T**_1_** state, that is, the reorganization energy increases significantly. In particular, complex **3** has a significant structural distortion due to one branch of the N^N ligand is replaced with the diphenylphosphine group, then the reorganization energy of complex **3** reaches 15,713.9cm^−1^.

In order to deeply investigate the origin of the reorganization energy, we divide the reorganization energy into a low-frequency vibration region (*ω* ≤ 1000cm^−1^) and a high-frequency vibration region (*ω* > 1000cm^−1^), respectively. It is verified that the increased reorganization energy mainly comes from low-frequency vibrations. The three larger vibrational modes of complexes **1**, **3** and **4** that contribute largely to reorganization energy are derived from the low-frequency vibrational modes, and especially for complexes **2** and **3**, the vibrational modes of largest reorganization energies mainly came from the low-frequency modes below 200 cm^−1^ (see Figures S3–S6).

According to Figures S3–S6, the vibration modes that have the largest contribution to the reorganization energy are analyzed. Evidently, the vibration mode 273 cm^−1^ of complex **1** mainly comes from the out-plane bending vibration of the N^N ligand, especially the atoms and methyl near the Cu(I) ion. However, when the N^N ligand is replaced by the P^P ligand (complex **2**: 179cm^−1^), the vibrations of the P^P ligand and the N^N ligand are suppressed due to large steric effects, only the methyl groups on the N^N ligand have rocking vibration. For complex **3**, the vibration mode 87 cm^−1^ has the largest contribution to the reorganization energy, which is attributed to the rocking vibrations of the two benzene rings connected to the P atom in the N^P ligand. In addition, the reorganization energy of the largest vibration modes already reaches 1347 cm^−1^, and several of the reorganization energy levels of the vibration mode exceed 1000 cm^−1^, these are derived from the rocking vibration of the benzene ring directly connected to the P atom in the N^P ligand. These results mean that the addition of the diphenylphosphine structure in the N^N ligand breaking of the original π conjugation, which led to a strengthening of vibrations from the benzene ring, finally result in a larger reorganization energy. The vibration with the largest contribution to the reorganization energy in complex **4** (163 cm^−1^) is mainly derived from the rocking vibration of the benzene ring connected to the O atom in the P^P ligand (independent P), and the reorganization energy also reaches 1333 cm^−1^.

As shown in Table 6, the values of the temperature-dependent term *μ*_1_ of complex **1** and **2** below 200 cm^−1^(**1**: *μ*_1_ = −2011 cm^−1^, **2**: *μ*_1_ = −712 cm^−1^) are negative and can increase the effective energy gap. To complexes **3** and **4**, the values of *μ*_1_ are positive, and the values of the temperature-dependent term *μ*_2_ of the high energy region are also positive, both of them reduce the effective energy gap, and as a result, bring about a sharp increase in *k*_nr_.

In summary, for complex **1**, although the phosphorescent radiative decay rate is calculated by Equation (4), considering the weak spin–orbit coupling between the T_1_ and S_0_ states, the excitons of the T_1_ state cannot accomplish phosphorescence emission. In spite of the lower proportion of MLCT in complex **2**, effective spin–orbit coupling matrix element, large singlet transition moments that can be borrowed, as well as the highest triplet excitation energy, result in the fastest radiative decay rate. The addition of the P^P ligand can effectively suppress the out-plane bending vibration of the N^N ligand, reduce the structural distortion between the excited state of the ground state, hence a smaller non-radiative decay rate was obtained, and finally, complex **2** has the highest quantum yield. Although complex **3** has a similar MLCT proportion to complex **2**, it has a lower *k*_r_ ascribing to the lower matching between the effective spin–orbit coupling and the larger oscillator strength (i.e., the small singlet transition moments that can be borrowed). At the same time, because of the addition of diphenylphosphine in the N^P ligand, the π conjugation of the original N^N ligand is broken, which results in a significant vibration of the benzene ring connected to the P atom in the N^P ligand. The *k*_nr_ of complex **3** is faster with the increase in reorganization energy, so complex **3** has a lower quantum yield than complex **2**. For complex **4**, with the presence of an un-chelated P atom, the contribution of the benzene ring, which is connected to the dangling P atom, to the reorganization energy is increased, so the quantum yield of complex **4** is lower than that of complex **2**.

## 4. Conclusions

In this paper, DFT/TDDFT methods are used to study the ground- and excited-state structures of a series of Cu(I) complexes bearing the 2,9-dimethyl-1,10-phenanthroline and bis [2-(diphenylphosphino)phenyl]ether ligand. The radiative decay rate, the factors affecting the non-radiative decay rate and the effects of different ligand structures from N^N to P^P ligand, including of mixed ligands, on the quantum efficiency are illustrated from the perspective of theoretical calculation. The results confirm that although the N^N homo-coordination complex has the highest metal participation in the transition process, the borrowable singlet transition moment is small, ascribing to the single MLCT transition, resulting in an unsatisfactory phosphorescent radiative decay. The addition of the P^P ligand can effectively raise the HOMO energy level, reduce the participation of Cu(I) ion and heighten the proportion of LLCT in the transition process, thus enhancing the probability of borrowable singlet transition moments, which is beneficial to the radiative decay. Complex **2**, because of the fastest radiative decay rate and effective inhibition of the bending vibration of the N^N ligand, has the highest quantum yield. For complex **3**, due to the addition of the diphenylphosphine structure in the N^N ligand, the conjugated nature of the original N^N ligand is destroyed to some extent, resulting in an imbalance of the electron-donating and -accepting in the N^P ligand, and moreover, there is no large steric hindrance, allowing the rocking vibration of the benzene ring connected to the P atom in the N^P ligand to increase, resulting in the largest reorganization energy and the largest non-radiative decay rate.

In summary, due to the high degree of coordination of electron-donating and electron-accepting between the N^N and P^P ligands and the perfect spatial chelation structure, complex **2** has a higher radiative decay rate and a lower non-radiative transition rate, that is, the hetero-coordinated structure with the N^N ligand and the P^P ligand has better applicable prospects for phosphorescent materials. In this paper, through the study of this series of Cu(I) complexes, the effects of the N^N and P^P ligands in Cu(I) complexes on the electronic structure and light-emitting properties are explained in depth, and the regulating effect of homo-leptic and hetero-leptic ligands on the phosphorescent emitting properties of Cu(I) complexes are scrutinized, which provides a valuable theoretical basis and reference.

## Figures and Tables

**Table 1:** Calculated *E*(S_1_) using various functionals and LANL2DZ/6–31G(d) basis set in CH_2_Cl_2_ media for complex **1** and **2**.

	BLYP (0%)	MPWLY P1M (5%)	TPSSH (10%)	B3LYP (20%)	PBE0 (25%)	MPW1 B95 (31%)	BMK (42%)	M06-2x (54%)
HF%	0	5	10	20	25	31	42	54
1 *E*(S_1_)/eV	1.9432	2.0863	2.2468	2.6259	2.7508	3.0825	3.3242	3.9777
2 *E*(S_1_)/eV	2.3308	2.5202	2.7997	3.1605	3.3400	3.5679	3.8770	4.2369

Note: Hartree–Fock percentage is listed in the parentheses.

**Table 2:** Calculated natural population analysis in S_0_ and T_1_ states (unit in e) by PBE0/[LANL2DZ-ECP/6-31G(d)].

Complex	S_0_			T_1_			S_0_-T_1_		
	Cu	Ligand 1	Ligand 2	Cu	Ligand 1	Ligand 2	Cu	Ligand 1	Ligand 2
**1**	0.5615	0.2193	0.2193	0.5617	0.2192	0.2192	0.0002	0.0001	0.0001
**2**	0.3633	0.2262	0.4105	0.3677	0.1973	0.4350	0.0044	0.0289	−0.0245
**3**	0.2257	0.3217	0.4526	0.3076	0.2809	0.4116	0.0819	0.0408	0.0410
**4**	0.5070	0.2919	0.2011	0.5149	0.2939	0.1918	0.0079	−0.0020	0.0093

**Table 3:** IPs, EAs, extraction potentials, and reorganization energies for complexes (unit in eV).

	IP (v)	IP (a)	HEP	EA (v)	EA (a)	EEP	*λ*_hole_	*λ*_electron_
**1**	9.55	9.16	8.88	3.28	3.37	3.45	0.67	0.16
**2**	9.03	8.70	8.42	2.94	3.18	3.41	0.61	0.46
**3**	8.94	8.55	8.16	2.83	3.08	3.33	0.77	0.50
**4**	8.81	8.34	7.72	2.18	2.33	2.51	1.09	0.33

**Table 4:** Emission spectra obtained by the TDDFT method in CH_2_Cl_2_ for studied complexes, together with experimental values.

	Electronic Transition	$\lambda_{max}^{emm}\;(\mathrm{nm})$	Configuration (%)	Assignment
**1**	T_1_→S_0_	782.9/730expt	H→L (98.0)	ML_N_L_N_CT
			H→L+4 (2.0)	ML_N_L_N_CT
**2**	T_1_→S_0_	612.7/570expt	H→L (92.5)	ML_N_CT/L_P_L_N_CT
			H−1→L (2.9)	ML_N_CT/L_P_L_N_CT
**3**	T_1_→S_0_	725.7/608expt	H→L (72.0)	ML_N_CT/L_P_L_N_CT
			H−4→L (19.5)	ML_N_CT/L_P_L_N_CT
			H−3→L (4.5)	ML_N_CT/L_P_L_N_CT
**4**	T_1_→S_0_	647.3	H→L (89.8)	L_P1_L_P2_CT

**Table 5:** Calculated excited triplet energy *E*(T_1_) and *k*_r_ based on the T_1_ geometry by TD PBE0//UPBE0/6-31G(d)&LAN2DZ.

	E(T_1_) (cm^−1^)	*k*_r_^x^ (s^−1^)	*k*_r_^y^ (s^−1^)	*k*_r_^z^ (s^−1^)	*k*_r_^avg^ (s^−1^)	*k*_r_^exp^ (s^−1^)
**1**	12,773	2074	142	2075	1430	4444
**2**	16,322	12,413	16,300	12,413	13,708	10,490
**3**	13,780	3302	10,239	3302	5614	17,143
**4**	15,450	10,506	47	10,506	7020	0

**Table 6:** Calculated spin–orbit coupling matrix, emission energy Δ*E*_00_, reorganization energy *λ*, Huang–Rhys factor (*S*) and the parameters related to *k*_nr_. (Energy unit in cm^−1^, rate unit in s^−1^).

	<T_1_|SOC|S_0_>^2^	Δ*E*_00_	λ_1_	μ_1_	μ_2_	n_M1_	n_M2_	λ_M_	λ_L_	λ	S_lf_	S_M_	S	*k*_nr_	*k*_nr_ (expt)
**1**	0.014	17,781	2141	−2011	185	12	11	783	2735	3518	41.14	0.53	0.4176	2.8 × 10^−5^	
**2**	4777	20,997	2977	−712	567	12	11	1378	3073	4451	33.96	0.93	34.89	9469.6	5.94 × 10^4^
**3**	1603	19,243	13,769	442	853	3	3	2267	13,447	15,714	254.28	1.55	255.82	1.2 × 10^11^	1.41 × 10^6^
**4**	497	25,918	12,409	416	1119	9	8	1371	12,182	13,553	66.62	0.97	69.59	8.8 × 10^5^	

## Data Availability

The data presented in this study are available on request from the corresponding author.

## Supplementary Materials

The following supporting information can be downloaded at: https://www.mdpi.com/article/10.3390/ma15207253/s1. Table S1: absorption spectra and emission wavelengths at the optimized S0 geometries for complexes **1**, **2**, **3** in PCM solution (CH_2_Cl_2_) together with the experimental values; Table S2: selected bond lengths (Å), bond angles (°) at the optimized S0 geometries for complexes **1**–**3** by PBE0/LANL2DZ&6-31G(d); Table S3: selected bond lengths (Å), bond angles (°) and dihedral angles (°) at optimized S0 and T1 geometries for complexes by PBE0/LANL2DZ&6-31G(d); Table S4: Natural charge population in S0 and T1 on the N and the P atoms of the complexes by using NBO method at the PBE0/LANL2DZ and 6-31 G(d) level; Table S5: Frontier molecular orbital (FMO) energies (eV) and compositions (%) of different fragments in the ground state for the complex. under considering solvent effect with DCM (Only FMO components contributing more than 10% are counted); Table S6: absorption spectra obtained by the TDDFT method in the CH_2_C_l2_ for these complexes, together with experimental values; Table S7: Frontier molecular orbital energies (eV) and compositions (%) of different fragments in the T1 state for the complexes. Table S8: the SOC matrix elements and oscillator strength of complex **1** at the optimized T1 state geometry by BDF package; Table S9: the SOC matrix elements and oscillator strength of complex **2** at the optimized T1 state geometry by BDF package; Table S10: the SOC matrix elements and oscillator strength of complex **3** at the optimized T1 state geometry by BDF package; Table S11: the SOC matrix elements and oscillator strength of complex **4** at the optimized T1 state geometry by BDF package; Figure S1: superimposed structures of S0 (green) and T1 (red) states of studied complexes; Figure S2: electronic density contours of the frontier orbitals in the T1 state for the complexes studied; Figure S3: diagrammatic illustration of the displacement vectors of the selected vibrational normal modes with the largest reorganization energies of complex **1**; Figure S4: diagrammatic illustration of the displacement vectors of the selected vibrational normal modes with the largest reorganization energies of complex **2**; Figure S5: diagrammatic illustration of the displacement vectors of the selected vibrational normal modes with the largest reorganization energies of complex **3**; Figure S6: siagrammatic illustration of the displacement vectors of the selected vibrational normal modes with the largest reorganization energies of complex **4**.
