# Supplementary material for: A Theoretical Investigation into the Homo- and Hetero-leptic Cu(I) Phosphorescent Complexes Bearing 2,9-dimethyl-1,10-phenanthroline and bis [2-(diphenylphosphino)phenyl]ether Ligand
Source: Materials (Basel). 2022 Oct 17;15(20):7253. doi: 10.3390/ma15207253 (PMC9608084; doi:10.3390/ma15207253)
Supplement: Supplementary file 1 [file materials-15-07253-s001.zip › materials-1972768-supplementary.pdf]

Supporting Information

The radiative transition rate is derived from Einstein's spontaneous emission coefficient and can be expressed as follows:

$$k_r^{\alpha}(\mathrm{T}_1 \rightarrow \mathrm{S}_0) = \frac{\eta^3 E(\mathrm{T}_1)^3}{1.5}\left\{\sum_n \frac{\left\langle T_1^{\alpha}\left|\hat{H}_{SOC}\right|\mathrm{S}_\mathrm{n}\right\rangle}{E(\mathrm{S}_\mathrm{n})-E(\mathrm{T}_1)} \times \left(\frac{f_\mathrm{n}}{E(\mathrm{S}_\mathrm{n})}\right)^{1/2}\right\}^2 \quad (1)$$

$$k_{r,avg}^{RT}(T_1 \rightarrow S_0) = \frac{1}{3}\sum_{\alpha} k_r^{\alpha} \quad (1a)$$

$$H_{SOC} = \sum_i \frac{1}{4\pi\varepsilon_0}\frac{Ze^2}{2m_e^2c^2}\frac{1}{r^3} l_i s_i = \zeta\sum_i l_i s_i \quad (1b)$$

In which $\left\langle \mathrm{T}_1\left|\hat{H}_{SOC}\right|\mathrm{S}_\mathrm{n}\right\rangle$ is the SOC matrix element of T_1-S_n, η is the refractive index of the medium (η of CH2Cl2 is taken to be 1.4244). *E(Tm)* and *E(Sn)* are the energies of the mth triplet and nth singlet states, respectively; f_n is the oscillator strength; ζ is the single electron spin orbit coupling constant. H_{SOC} is the Hamiltonian,, *ε0*, *Z*, *mε*, *c*, *r*, *li*, *si* are the dielectric constant in vacuum, the number of nuclear charges, the resting mass of electrons, the speed of light, the distance between electrons and nuclei, the angular momentum operator, and the spin operator.

The non-radiative transition rate knr can be estimated by equation (2), in which the contributions of the vibrations to *knr* from low frequency region and high frequency region are separately considered{hf and lf designate the high-frequency ($1700>\omega_{hf}>1000$ cm^{-1}) and low-frequency modes ($\omega_{lf}\leq1000$ cm^{-1}), respectively}. When the suming (S_{lf}) of the Huang–Rhys factors for the low frequency modes is larger, $S_{lf}>1$, means that lf modes should be treated in the strong coupling limit. In

the weak coupling limit, it requires the Huang–Rhys factor S<1 or $\hbar\omega_M >> k_BT$, therefore knr is evaluated with lf modes treated in the strong coupling limit and hf modes in the weak coupling limit for studied complexes.

$$k_{nr}(\mathrm{T_1 - S_0}) = \frac{2\pi\langle \mathrm{T_1}|H_{SOC}|\mathrm{S_m}\rangle^2}{\hbar^2}\left[2\pi\hbar^2(D_1^2 + P^2)\right]^{-1/2} exp\left[-\frac{(\Delta E_{00} - n_M\hbar\omega_M - \lambda_1 - \mu)}{2\pi\hbar^2(D_1^2 + P^2)}\right] exp\left(-S_M\frac{S_M^{n_M}}{n_M!}\right) \quad (2)$$

$$b_j = \frac{\omega_j^T}{\omega_j^S} \quad (2a)$$

$$\hbar^2 D_1^2 = \sum_{j\in lf} S_j\left(\frac{\hbar\omega_j^S}{b_j}\right)^2 coth\frac{\hbar\omega_j^T}{2k_BT} \quad (2b)$$

$$\hbar^2 P^2 = \frac{1}{2}\sum_{j\in lf}\left[\hbar\omega_j^S\frac{1 - b_j^2}{b_j} coth\frac{\hbar\omega_j^T}{2k_BT}\right]^2 \quad (2c)$$

$$\lambda_M = \sum_{j\in lf} S_j\,\hbar\omega_j \quad (2d)$$

$$\hbar\omega_M = \frac{\lambda_M}{S_M} \quad (2e)$$

$$\lambda_1 = \sum_{j\in lf}\frac{S_j\hbar\omega_j^S}{b_j} \quad (2f)$$

$$\mu = \frac{1}{2}\sum_{j\in lf}\hbar\omega_j^S\frac{1 - b_j^2}{b_j} coth\frac{\hbar\omega_j^T}{2k_BT} \quad (2g)$$

$$n_M = \frac{\Delta E_{00} - \lambda_1 - \mu}{\hbar\omega_M} \quad (2h)$$

$$S_M = \sum_{j \in lf} S_j \tag{2i}$$

Where, ΔE_{00} is the zero-point energy difference between the T_1 and S_0 states, n_M is the number of quanta of the effective high frequency mode ħω, the quotient is corrected to the smaller integer. S_j is the Huang–Rhys factor corresponding to the equilibrium displacement of the *j*th normal mode ω_j, S_M is the sum of the Huang–Rhys factors in high frequency mode.

Table S1. Absorption spectra and emission wavelengths at the optimized S_0 geometries for complexes **1, 2, 3** in PCM solution (CH_2Cl_2) together with the experimental values.

		**Absorption spectrum （Oscillator strength）**		**Emission spectrum**
**1**	B3LYP	440.6（0.1802）		794.4
	PBE0	450.3（0.0005）		782.9
	**expt**	**457**		**730**
**2**	B3LYP	397.5（0.0583）		623.2
	PBE0	376.5（0.0732）		612.7
	**expt**	**383**		**570**
**3**	B3LYP	393.2（0.0007）	291.6（0.0984）	702.0
	PBE0	347.2（0.0173）	281.9（0.1123）	725.7
	**expt**	**350**	**282**	**608**

Table S2 Selected bond lengths (Å), bond angles (°) at the optimized S_0 geometries for complexes 1-3 by PBE0/LANL2DZ&6-31G(d).

	**B3LYP**	**PBE0**	**Expt**		**B3LYP**	**PBE0**	**Expt**		**B3LYP**	**PBE0**	**Expt**
**1**	S_0	S_0	S_0	**2**	S_0	S_0	S_0	**3**	S_0	S_0	S_0
**Cu-N$_1$**	2.1156	2.0948	2.0403	**Cu-N$_1$**	2.1921	2.1635	2.0843	**Cu-N$_1$**	2.2860	2.2340	2.1932
**Cu-N$_2$**	2.1158	2.0948	2.0349	**Cu-N$_2$**	2.2016	2.1767	2.1041	**Cu-P$_1$**	2.4061	2.3691	2.2799
**Cu-N$_3$**	2.1158	2.0948	2.0181	**Cu-P$_1$**	2.4061	2.3619	2.2691	**Cu-P$_2$**	2.4167	2.3701	2.3000
**Cu-N$_4$**	2.1157	2.0948	2.0591	**Cu-P$_2$**	2.4276	2.3746	2.2728	**Cu-P$_3$**	2.4577	2.3972	2.3169
**N$_1$-Cu-N$_2$**	80.49	80.62	82.42	**N$_1$-Cu-N$_2$**	77.81	78.12	80.88	**N$_1$-Cu-P$_1$**	81.26	81.13	84.32
**N$_3$-Cu-N$_4$**	80.49	80.62	82.54	**P$_1$-Cu-P$_2$**	113.92	113.12	116.44	**P$_2$-Cu-P$_3$**	111.62	111.43	108.90
**N$_1$-Cu-N$_3$**	125.64	125.74	127.02	**N$_1$-Cu-P$_1$**	118.47	118.69	115.22	**N$_1$-Cu-P$_2$**	124.32	124.59	122.30
**N$_1$-Cu-N$_4$**	125.65	125.37	128.14	**N$_1$-Cu-P$_2$**	107.74	108.24	107.76	**N$_1$-Cu-P$_3$**	104.72	104.86	106.05
**N$_2$-Cu-N$_3$**	125.61	125.37	128.69	**N$_2$-Cu-P$_1$**	113.81	113.66	110	**P$_1$-Cu-P$_2$**	117.65	117.35	119.63
**N$_2$-Cu-N$_4$**	125.64	125.74	113.29	**N$_2$-Cu-P$_2$**	120.31	120.71	121.43	**P$_1$-Cu-P$_3$**	113.89	113.32	113.49
**DHA1**	89.99	89.64	81.34	**DHA**	82.43	82.36	82.31	**DHA**	83.09	83.35	85.35

Table S3 Selected bond lengths (Å), bond angles (°) and dihedral angles (°) at optimized S_0 and T_1 geometries for complexes by PBE0/LANL2DZ&6-31G(d).

	**1**				**2**				**3**				**4**		
	S_0	T_1	Exp/ S_0		S_0	T_1	Exp/ S_0		S_0	T_1	Exp/ S_0		S_0	T_1	Exp/ S_0
**Cu-N$_1$**	2.0948	2.0064	2.0403	**Cu-N$_1$**	2.1635	1.9705	2.0843	**Cu-N$_1$**	2.2340	2.0958	2.1932	**Cu-P$_1$**	2.3668	2.3579	2.2686
**Cu-N$_2$**	2.0948	2.0065	2.0349	**Cu-N$_2$**	2.1767	2.0647	2.1041	**Cu-P$_1$**	2.3691	2.4309	2.2799	**Cu-P$_2$**	2.3764	2.3793	2.2782
**Cu-N$_3$**	2.0948	2.0065	2.0181	**Cu-P$_1$**	2.3619	2.4381	2.2691	**Cu-P$_2$**	2.3701	2.4042	2.3000	**Cu-P$_3$**	2.3653	2.3515	2.2718
**Cu-N$_4$**	2.0948	2.0066	2.0591	**Cu-P$_2$**	2.3746	2.4806	2.2728	**Cu-P$_3$**	2.3972	2.4550	2.3169	**Cu-P$_4$**	**4.1866**	**4.6674**	**3.9576**
**N$_1$-Cu-N$_2$**	80.62	83.53	82.42	**N$_1$-Cu-N$_2$**	78.12	83.76	80.88	**N$_1$-Cu-P$_1$**	81.13	80.10	84.32	**P$_1$-Cu-P$_2$**	113.14	113.80	113.87
**N$_3$-Cu-N$_4$**	80.62	83.53	82.54	**P$_1$-Cu-P$_2$**	113.12	104.99	116.44	**P$_2$-Cu-P$_3$**	111.43	112.34	108.90	**P$_3$-Cu-P$_4$**	101.58	100.68	104.85

**N_1-Cu-N_3**	125.74	140.78	127.02	**N_1-Cu-P_1**	118.69	136.36	115.22	**N_1-Cu-P_2**	124.59	129.18	122.30	**P_1-Cu-P_3**	124.38	125.27	121.85
**N_1-Cu-N_4**	125.37	109.76	128.14	**N_1-Cu-P_2**	108.24	98.96	107.76	**N_1-Cu-P_3**	104.86	107.78	106.05	**P_1-Cu-P_4**	97.98	99.82	96.82
**N_2-Cu-N_3**	125.37	109.77	128.69	**N_2-Cu-P_1**	113.66	110.50	110.00	**P_1-Cu-P_2**	117.35	112.24	119.63	**P_2-Cu-P_3**	120.05	118.80	121.33
**N_2-Cu-N_4**	**125.74**	140.75	**113.29**	**N_2-Cu-P_2**	120.71	124.13	121.43	**P_1-Cu-P_3**	113.32	110.39	113.49	**P_2-Cu-P_4**	84.85	82.64	84.50
**DHA1**	**89.64**	60.56	**81.34**	**DHA1**	82.36	68.52	82.31	**DHA1**	83.35	83.44	85.35	**DHA1**	82.33	80.01	82.70
**DHA2**	0.09	4.11		**DHA2**	3.70	0.60		**DHA2**	2.81	10.37		**DHA2**			

（DHA1: the dihedral angle (DHA) between the N-Cu-N and P-Cu-P plane.DHA2 notes the dihedral angle between the pyridyl and azole plane.）

Table S4 Natural charge population in S_0 and T_1 on the N and the P atons of the complexes by using NBO method at the PBE0/ PBE0/LANL2DZ & 6-31 G(d) level.

		**N_1**	**N_2**	**N_3**	**N_4**
**1**	S_0	-0.5159	-0.5159	-0.5159	-0.5159
	T_1	-0.5103	-0.5103	-0.5103	-0.5103
**2**		**N_1**	**N_2**	**P_1**	**P_2**
	S_0	-0.5121	-0.5040	0.9307	0.9412
	T_1	-0.5347	-0.5150	0.9508	0.9784
**3**		**N_1**	**P_1**	**P_2**	**P_3**
	S_0	-0.5125	0.9500	0.9538	0.9639
	T_1	-0.5409	0.9817	0.9479	0.9558
**4**		**P_1**	**P_2**	**P_3**	**P_4**
	S_0	0.8626	0.86300	0.8607	0.9411
	T_1	0.8646	0.8649	0.8618	0.9509

Table S5 Frontier molecular orbital（FMO） energies (eV) and compositions (%) of different fragments in the **ground state** for the complex. under considering solvent effect with DCM (Only FMO components contributing more than 10% are counted)

	**Energy/Ev**	**MO composition（%）**			**Assign**
**1**		**Cu**	**N^N**	**N^N**	
L+5	-0.736	2.7	48.6	48.6	π*(N^N)
L+4	-0.755	2.2	48.9	48.9	π*(N^N)
L+3	-1.954	0	50	50	π*(N^N)
L+2	-1.973	0.6	49.7	49.7	π*(N^N)
L+1	-2.152	3.4	48.2	48.3	π*(N^N)
L	-2.154	3.4	48.4	48.2	π*(N^N)
H	-6.141	70.5(68.2*dxy*)	14.8	14.8	d(Cu)+π(N^N)
H-1	-6.157	70.8(68.4 *dxz*)	14.6	14.6	d(Cu)+π(N^N)
H-2	-6.902	87.4(84.8 *dyz*)	6.3	6.3	d(Cu)
H-3	-7.049	88(64.0 *dz2*+23.9 *dx2-y2*)	6	6	d(Cu)
H-4	-7.136	95(25.4 *dz2*+67.6 *dx2-y2*)	2.5	2.5	d(Cu)
H-5	-7.14	4.2	47.9	47.9	π(N^N)
**2**		**Cu**	**N^N**	**POP**	
L+6	-0.751	3.6	82.7	13.7	π*(N^N +POP)
L+5	-0.929	1.8	3.5	94.8	π*(N^N+POP)
L+4	-1.05	2.9	2.3	94.9	π*(POP)
L+3	-1.089	2	3.1	94.9	π*(POP)
L+2	-1.205	1.7	3	95.3	π*(POP)
L+1	-1.958	0.4	97.6	2	π*(N^N)
L	-2.145	3.6	92.3	4.1	π*(N^N)

H	-6.267	36.2（29.4 *dx2-y2*）	6.4	57.5	d(Cu)+π(POP)
H-1	-6.899	44.8（15.5*dz2*+18.5 *dxy*）	9.8	45.4	d(Cu)+π(POP)
H-2	-6.91	60（30.4 *dxz*+25.8 *dyz*）	26.2	13.9	d(Cu)+π(N^N+POP)
H-3	-7.031	16	17.8	66.1	d(Cu)+π(N^N+POP)
H-4	-7.103	2.5	75.2	22.3	π(N^N+POP)
H-5	-7.43	9.3	15.3	75.4	π(N^N +POP)
H-6	-7.462	5.4	53.2	41.4	π(N^N +POP)
H-7	-7.483	3.2	7.2	89.6	π(POP)
H-8	-7.537	3.5	8.6	87.9	π(POP)
H-9	-7.605	3.4	15.1	81.5	π(N^N +POP)
H-11	-7.664	12.9	4.1	83.0	d(Cu)+π(POP)
H-15	-7.847	50.0（21.3 *dxz*+15.9 *dyz*+10.0 *dx2-y2*）	4.1	45.9	d(Cu)+π(POP)
H-16	-7.911	68.4	3.3	28.2	d(Cu)+π(POP)
H-17	-7.953	58.3	4.7	37.0	d(Cu)+π(POP)
H-18	-8.061	14.9	2.3	82.7	d(Cu)+π(POP)
**3**		**Cu**	**NP**	**POP**	
L+5	-0.994	2.9	37.6	59.5	π*(NP+POP))
L+4	-1.034	2.1	44	53.9	π*(NP+POP)
L+3	-1.059	3.2	34.3	62.4	π*(NP+POP)
L+2	-1.167	6.3	15.1	78.6	π*(NP+POP)
L+1	-1.232	2.4	13.2	84.4	π*(NP+POP)
L	-2.129	0.3	98.5	1.2	π*(NP)
H	-6.31	35.7（14.6*dxy*）	32	32.3	d(Cu)+π(NP+POP)
H-1	-6.457	31.4（11.7*dx2-y2*）	26.3	42.4	d(Cu)+π(NP+POP)
H-2	-6.994	31.6（18.6*dz2*）	14.3	54	d(Cu)+π(NP+POP)
H-3	-7.092	10.9	6.9	82.2	d(Cu)+π(POP)

H-4	-7.207	3	89.2	7.7	π(NP)
H-5	-7.436	1.1	58.8	40.1	π(NP+POP)
**4**		**Cu**	**POP1**	**POP2**	
L+6	-0.896	5.3	25.7	69	π*(POP1+POP2)
L+5	-1.008	1.2	41.1	57.6	π*(POP1+POP2)
L+4	-1.046	3.9	81.3	14.8	π*(POP1+POP2)
L+3	-1.092	3.6	69	27.3	π*(POP1+POP2)
L+2	-1.149	4	27.1	68.9	π*(POP1+POP2)
L+1	-1.211	8.6	71.5	19.9	π*(POP1+POP2)
L	-1.353	5.5	82.7	11.8	π*(POP1+POP2)
H	-6.317	2.5	2.3	95.2	π(POP2)
H-1	-6.55	26.1 （11.2 *dyz*）	71.2	2.7	d(Cu)+π(POP1)
H-2	-6.609	23.2	18.9	58	d(Cu)+π(POP1+POP2)
H-3	-7.033	6	10	84.1	π(POP1+POP2)
H-4	-7.1	4.4	93.8	1.9	π(POP1)
H-5	-7.33	4.6	24	71.4	π(POP1+POP2)

Table S6. Absorption spectra obtained by the TDDFT method in the CH_2Cl_2 for these complexes, together with experimental values.

	**Electronic transition**	λ_{max}^{abs} **(nm)**	***f***	**Configuration（%）**	**Assignment**
**1**	$S_0 \rightarrow S_1$	450.3/457^{expt}	0.0005	H→L(54.1%)	ML_NL_NCT
				H-1→L+1(44.2%)	ML_NL_NCT
	$S_0 \rightarrow S_2$	440.8	0.0000	H→L+1(52.0%)	ML_NL_NCT
				H-1→L (46.1%)	ML_NL_NCT
	$S_0 \rightarrow S_3$	417.21/457^{expt}	0.1926	H-1→L+1(56.2%)	ML_NL_NCT
				H→L(44.2%)	ML_NL_NCT

	$S_0 \rightarrow S_{29}$	277.3	0.0545	H-1 $\rightarrow$ L+5(62.7%)	ML_NL_NCT
				H $\rightarrow$ L+4(32.0%)	ML_NL_NCT
**2**	$S_0 \rightarrow S_1$	376.5/383^{expt}	0.0732	H $\rightarrow$ L (92.5%)	ML_NCT /L_PL_NCT
				H-2 $\rightarrow$ L (3.4%)	ML_NCT /L_PL_NCT
	$S_0 \rightarrow S_{10}$	289.6	0.0746	H $\rightarrow$ L+3(72.0%)	ML_NL_PCT
				H-4 $\rightarrow$ L+1(9.7%)	L_PL_NCT
				H $\rightarrow$ L+2(5.1%)	ML_PCT
				H-6 $\rightarrow$ L (3.4%)	L_PL_NCT
				H-3 $\rightarrow$ L+1(2.9%)	ML_NCT /L_PL_NCT
	$S_0 \rightarrow S_{15}$	272.6	0.1543	H-15 $\rightarrow$ L（23.1%)	ML_NCT /L_PL_NCT
				H-6 $\rightarrow$ L（15.7%)	L_PL_NCT
				H-17 $\rightarrow$ L（11.5%)	ML_NCT /L_PL_NCT
				H-16 $\rightarrow$ L（8.0%)	ML_NCT /L_PL_NCT
				H-4 $\rightarrow$ L+1（8.0%)	L_PL_NCT
				H-5 $\rightarrow$ L（7.2%)	ML_NCT /L_PL_NCT
				H-3 $\rightarrow$ L+1（6.5%)	ML_NCT /L_PL_NCT
				H $\rightarrow$ L+5（3.4%)	ML_NCT /L_PL_NCT
				H-9 $\rightarrow$ L（2.9%)	ML_NCT /L_PL_NCT
				H-18 $\rightarrow$ L（2.4%)	ML_NCT /L_PL_NCT
				H-11 $\rightarrow$ L（2.4%)	ML_NCT /L_PL_NCT
**3**	$S_0 \rightarrow S_1$	375.2/350^{expt}	0.0007	H $\rightarrow$ L (87.1%)	ML_NCT /L_PL_NCT
				H-1 $\rightarrow$ L (10.6%)	ML_NCT /L_PL_NCT
	$S_0 \rightarrow S_2$	347.2/350^{expt}	0.0173	H-1 $\rightarrow$ L (87.1%)	ML_NCT /L_PL_NCT
				H $\rightarrow$ L (10.6%)	ML_NCT /L_PL_NCT
	$S_0 \rightarrow S_8$	281.9/282^{expt}	0.1123	H $\rightarrow$ L+3(60.5%)	ML_PCT /L_NL_PCT
				H $\rightarrow$ L+2(9.7%)	ML_PCT /L_NL_PCT

				H-1 → L+2(9.7%)	ML_PCT /L_NL_PCT
				H-1 → L+1 (6.5%)	ML_PCT
**4**	$S_0 \rightarrow S_1$	293.0	0.0505	H → L (89.8%)	$L_{P2}L_{P1}CT$
				H-1 → L (6.5%)	$ML_{P1}CT$ /$L_{P2}L_{P1}CT$
	$S_0 \rightarrow S_3$	286.4	0.1195	H → L+2(48.0%)	$L_{P2}L_{P1}CT$
				H → L+6(15.7%)	$L_{P2}L_{P1}CT$
				H → L+3(15.7%)	$L_{P2}L_{P1}CT$
				H → L+1(6.5%)	$L_{P2}L_{P1}CT$
				H-1 → L (4.5%)	$ML_{P1}CT$ /$L_{P2}L_{P1}CT$

Table S7 Frontier molecular orbital energies (eV) and compositions (%) of different fragments in **the T_1 state** for the complexes.

	Energy/eV	MO composition(%)			Assign
**1**		Cu	N^N1	N^N2	
**L+5**	-0.615	1.8	49.1	49.1	π*(N^N+ N^N)
**L+4**	-0.922	2.7	48.7	48.7	π*(N^N+ N^N)
**L+3**	-1.96	0.6	49.7	49.8	π*(N^N+ N^N)
**L+2**	-2.069	0.4	49.9	49.8	π*(N^N+ N^N)
**L+1**	-2.269	3.4	48.1	48.5	π*(N^N+ N^N)
**L**	-2.372	5	47.7	47.3	π*(N^N+ N^N)
**H**	-5.503	61.7	19.2	19.2	d(Cu)+π(N^N+ N^N)
**H-1**	-6.552	62.2	18.9	18.9	d(Cu)+π(N^N+ N^N)
**H-2**	-7.014	81.6	9.2	9.2	d(Cu)
**H-3**	-7.148	78.4	10.8	10.8	d(Cu)+π(N^N+ N^N)
**H-4**	-7.221	1.1	49.4	49.5	π(N^N+ N^N)
**H-5**	-7.308	23.6	38.2	38.2	d(Cu)+π(N^N+ N^N)
**2**		Cu	N^N	POP	

**L+5**	-0.885	1.5	2.3	96.2	π*(POP)
**L+4**	-1.046	1.9	3	95.1	π*(POP)
**L+3**	-1.086	3.6	2.9	93.5	π*(POP)
**L+2**	-1.208	1.6	3.9	94.6	π*(POP)
**L+1**	-1.998	0.6	97.2	2.2	π*(N^N)
**L**	-2.494	4.4	90.8	4.7	π*(N^N)
**H**	-5.75	39.4	13.2	47.3	d(Cu)+π(N^N+POP)
**H-1**	-6.682	57.9	23.4	18.8	d(Cu)+π(N^N+POP)
**H-2**	-6.892	42	13.3	44.7	d(Cu)+π(N^N+POP)
**H-3**	-7.003	6.5	1.9	91.6	π(POP)
**H-4**	-7.153	7.8	73.8	18.4	π(N^N+POP)
**H-5**	-7.37	19.4	58.5	22.1	d(Cu)+π(N^N+POP)
**3**		**Cu**	**NP**	**POP**	
**L+5**	-0.948	2	26	72	π*(NP+POP)
**L+4**	-0.978	3.4	51.4	45.2	π*(NP+POP)
**L+3**	-1.05	8.1	45.4	46.6	π*(NP+POP)
**L+2**	-1.178	3.4	6	90.7	π*(POP)
**L+1**	-1.221	3.8	14.4	81.8	π*(NP+POP)
**L**	-2.618	1	96.3	2.7	π*(NP)
**H**	-5.725	26.2	62.7	11.1	d(Cu)+π(NP+POP)
**H-1**	-6.274	33.2	5.2	61.6	d(Cu)+π(POP)
**H-2**	-6.882	44.7	14.3	40.9	d(Cu)+π(NP+POP)
**H-3**	-7.048	5.8	11.8	82.3	π(NP+POP)
**H-4**	-7.204	16.5	65.7	17.9	d(Cu) +π(NP+POP)
**H-5**	-7.484	2	50.4	47.6	π(NP+POP)
**4**		Cu	POP1	POP2	

**L+5**	-1.018	2.9	40.9	56.2	π*(POP1+POP2)
**L+4**	-1.047	3	84.5	12.5	π*(POP1+POP2)
**L+3**	-1.15	5.1	35.7	59.3	π*(POP1+POP2)
**L+2**	-1.195	9.1	69.4	21.6	π*(POP1+POP2)
**L+1**	-1.306	6.4	74.5	19	π*(POP1+POP2)
**L**	-1.721	0.8	2	97.2	π*(POP2)
**H**	-5.183	0.4	1.3	98.3	π(POP2)
**H-1**	-6.528	26.2	70.9	3	d(Cu)+π(POP1)
**H-2**	-6.592	25.2	20.8	53.9	d(Cu)+π(POP1+POP2)
**H-3**	-7.068	4.8	75.9	19.3	π(POP1+POP2)
**H-4**	-7.093	5.5	29.3	65.1	π(POP1+POP2)
**H-5**	-7.279	2.4	15.4	82.2	π(POP1+POP2)

Table S8. The SOC matrix elements and oscillator strength of complex **1** at the optimized T_1 state geometry by BDF package

**Sm**	$\mathbf{f_{Sm}}$	$\langle T_1^x \| H_{SOC} \| S_m \rangle$		$\langle T_1^y \| H_{SOC} \| S_m \rangle$	$\langle T_1^z \| H_{SOC} \| S_m \rangle$		$\langle T_1 \| H_{SOC} \| S_m \rangle$
		Re	Im	Im	Re	Im	
$\mathbf{S_1}$	0.0531	0.009616	-0.00045	-0.02076	0.009616	0.00045	0.01
$\mathbf{S_2}$	0	-41.3087	-0.01676	-0.00697	-41.3087	0.016755	27.54
$\mathbf{S_3}$	0.0097	0.012814	0.00446	-0.00823	0.012814	-0.00446	0.01
$\mathbf{S_4}$	0.0063	-0.0173	-86.4098	-0.05672	-0.0173	86.40978	57.63
$\mathbf{S_5}$	0.1599	0.024529	0.07705	-216.722	0.024529	-0.07705	72.29
$\mathbf{S_6}$	0.0011	0.057129	106.6301	0.215322	0.057129	-106.63	71.16
$\mathbf{S_7}$	0	125.4158	-0.14145	0.012995	125.4158	0.141448	83.61

**S_8**	0.0002	-0.03321	-0.05481	137.7643	-0.03321	0.054808	45.96
**S_9**	0.0005	-0.01339	-0.00771	-0.00882	-0.01339	0.007714	0.01
**S_{10}**	0.016	0.019102	44.06051	0.0808	0.019102	-44.0605	29.40
**SUM**	SOC/ΔE*(f/E) ^0.5	1.12E-08	-2.3E-06	-4.66495E-05	1.12E-08	2.33E-06	

Table S9. The SOC matrix elements and oscillator strength of complex **2** at the optimized T_1 state geometry by BDF package

**Sm**	**f_{Sm}**	$\langle T_1^x \| H_{SOC} \| S_m \rangle$		$\langle T_1^y \| H_{SOC} \| S_m \rangle$	$\langle T_1^z \| H_{SOC} \| S_m \rangle$		$\langle T_1 \| H_{SOC} \| S_m \rangle$
		Re	Im	Im	Re	Im	
**S_1**	0.0406	-79.4965	28.09261	-10.5854	-79.4965	-28.0926	59.74
**S_2**	0.0128	-67.1038	12.7852	9.735501	-67.1038	-12.7852	48.79
**S_3**	0.0632	-51.5906	-63.9985	136.0662	-51.5906	63.99847	100.16
**S_4**	0.0071	60.62225	63.56547	118.6371	60.62225	-63.5655	98.10
**S_5**	0.0169	13.56101	14.93093	136.8401	13.56101	-14.9309	59.06
**S_6**	0.0141	48.80035	-84.143	-60.9723	48.80035	84.14298	85.17
**S_7**	0.0194	159.7131	89.62804	271.2522	159.7131	-89.628	212.51
**S_8**	0.0469	-70.1672	-13.692	-183.102	-70.1672	13.69195	108.69
**S_9**	0.0334	-6.43982	18.8387	-14.4918	-6.43982	-18.8387	18.10
**S_{10}**	0.0249	6.169143	-2.42423	-9.20188	6.169143	2.424234	7.49
**SUM**	SOC/ΔE*(f/E)^0.5	-6.3E-05	1.42E-05	-9.20188	-6.3E-05	-1.4E-05	

Table S10. The SOC matrix elements and oscillator strength of complex **3** at the optimized T_1 state geometry by BDF package

**Sm**	f_{Sm}	$\langle T_1^x \| H_{SOC} \| S_m \rangle$		$\langle T_1^y \| H_{SOC} \| S_m \rangle$	$\langle T_1^z \| H_{SOC} \| S_m \rangle$		$\langle T_1 \| H_{SOC} \| S_m \rangle$
		Re	Im	Im	Re	Im	
S_1	0.0379	39.40749	3.981269	-40.8922	39.40749	-3.98127	40.04
S_2	0.0223	20.75149	3.487207	-20.9552	20.75149	-3.48721	21.01
S_3	0.0063	-14.2695	62.32052	-86.0439	-14.2695	-62.3205	71.30
S_4	0.0501	-82.7605	-30.0794	62.15567	-82.7605	30.07937	79.42
S_5	0.0552	-115.589	128.9329	-241.634	-115.589	-128.933	195.98
S_6	0.0449	243.8439	17.51099	-119.937	243.8439	-17.511	202.96
S_7	0.0507	53.98851	21.73239	-14.208	53.98851	-21.7324	43.531
S_8	0.0281	-10.5201	-2.93478	-17.7967	-10.5201	2.934776	13.21
S_9	0.0167	3.111329	10.2453	-9.22743	3.111329	-10.2453	10.21
S_{10}	0.0109	1.83997	7.173849	1.514274	1.83997	-7.17385	5.44
**SUM**	SOC/ΔE*(f/E)^0.5	2E-05	1.52E-05	-4.4E-05	2E-05	-1.5E-05	

Table S11. The SOC matrix elements and oscillator strength of complex **4** at the optimized T_1 state geometry by BDF package

**Sm**	f_{Sm}	$\langle T_1^x \| H_{SOC} \| S_m \rangle$		$\langle T_1^y \| H_{SOC} \| S_m \rangle$	$\langle T_1^z \| H_{SOC} \| S_m \rangle$		$\langle T_1 \| H_{SOC} \| S_m \rangle$
		Re	Im	Im	Re	Im	
S_1	0.0654	14.64482	22.97151	-2.6531	14.64482	-22.9715	19.05
S_2	0.0186	74.56597	16.19898	-31.2138	74.56597	-16.199	61.27

**S_3**	0.0156	-9.35883	0.434855	26.99434	-9.35883	-0.43486	15.24
**S_4**	0.007	21.06074	-44.0355	-72.0804	21.06074	44.03549	56.57
**S_5**	0.0439	15.08348	213.7523	79.22593	15.08348	-213.752	169.26
**S_6**	0.0479	-0.64971	47.84255	-57.4346	-0.64971	-47.8425	51.04
**S_7**	0.0109	9.288578	27.67726	168.3136	9.288578	-27.6773	75.57
**S_8**	0.0081	1.605931	28.95352	-44.2431	1.605931	-28.9535	34.08
**S_9**	0.0529	4.275035	0.258466	-7.85458	4.275035	-0.25847	5.47
**S_{10}**	0.0077	1.297024	-7.69371	1.700623	1.297024	7.693707	5.77
**SUM**	SOC/ΔE*(f/E)^0.5	1.47E-05	3.6E-05	4.99E-12	1.47E-05	-3.6E-05	

1	2	3	4
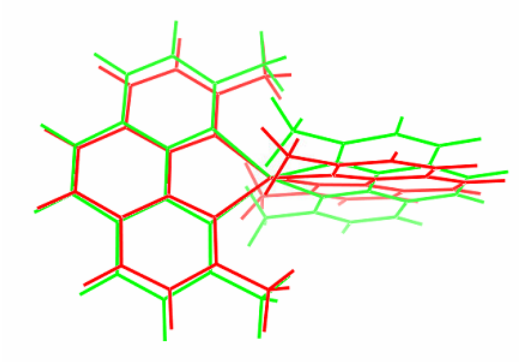	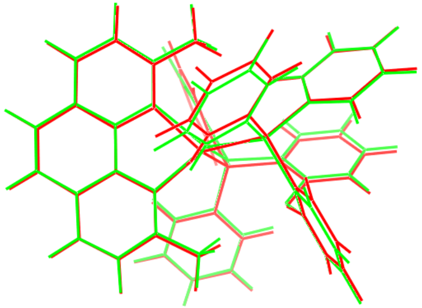	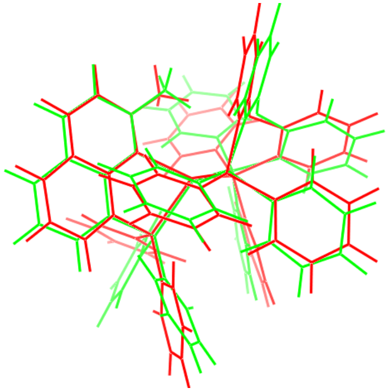	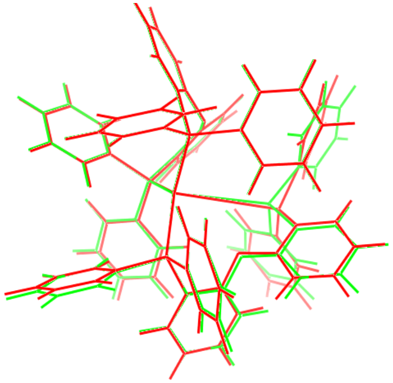
RMSD=0.575	RMSD =0.376	RMSD=1.026	RMSD=0.250

Figure S1. Superimposed structures of S_0 (green) and T_1 (red) states of studied complexes.

	HOMO-1	HOMO	LUMO	LUMO+1
1	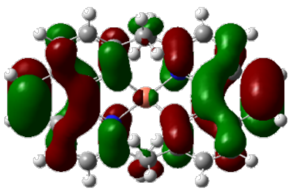	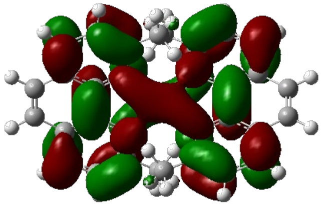	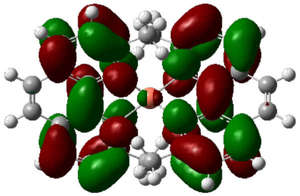	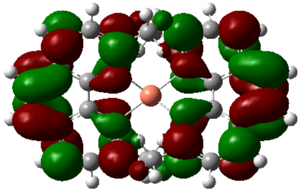

2

3

4

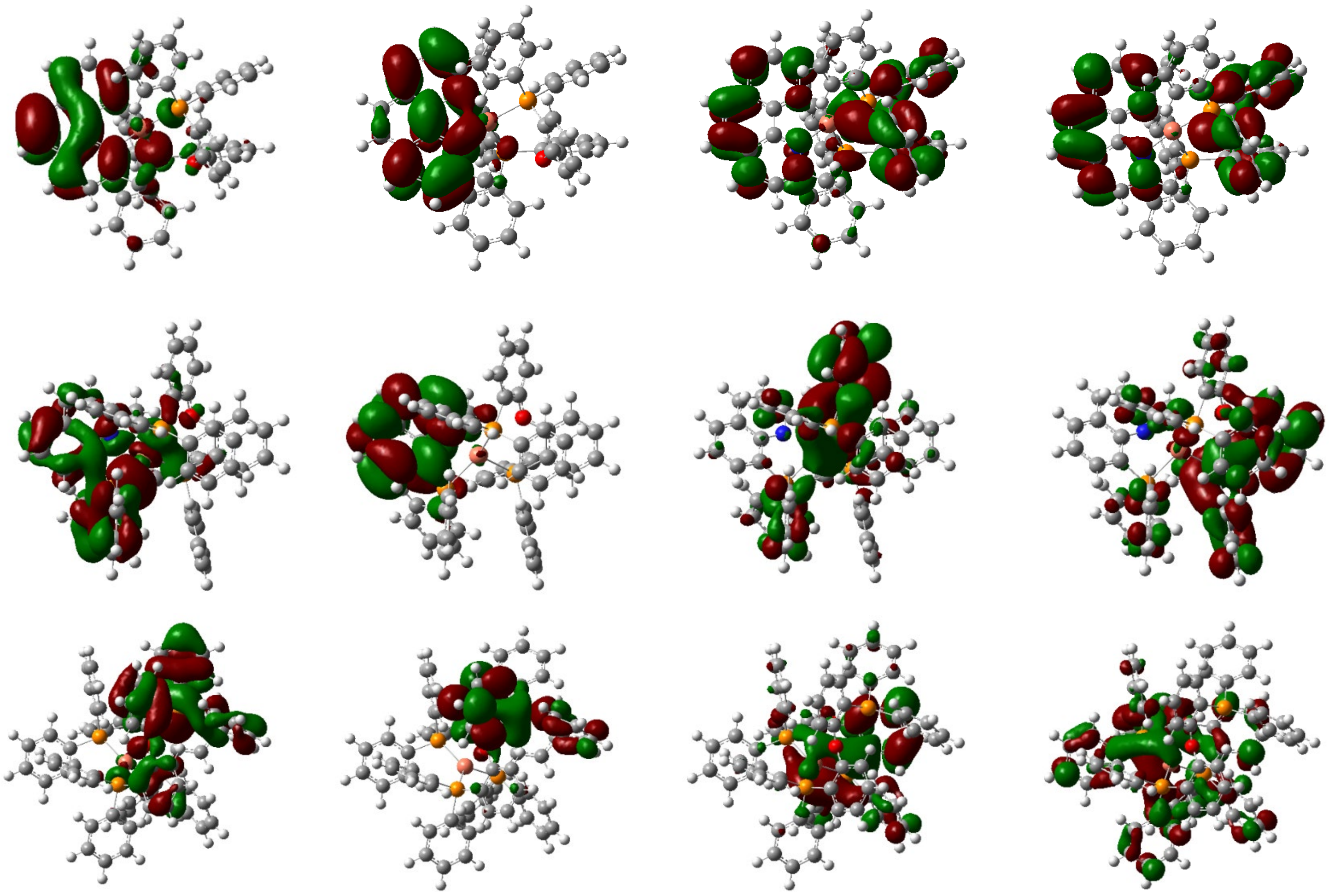

Figure S2. Electronic density contours of the frontier orbitals in the T_1 state for the complexes studied.

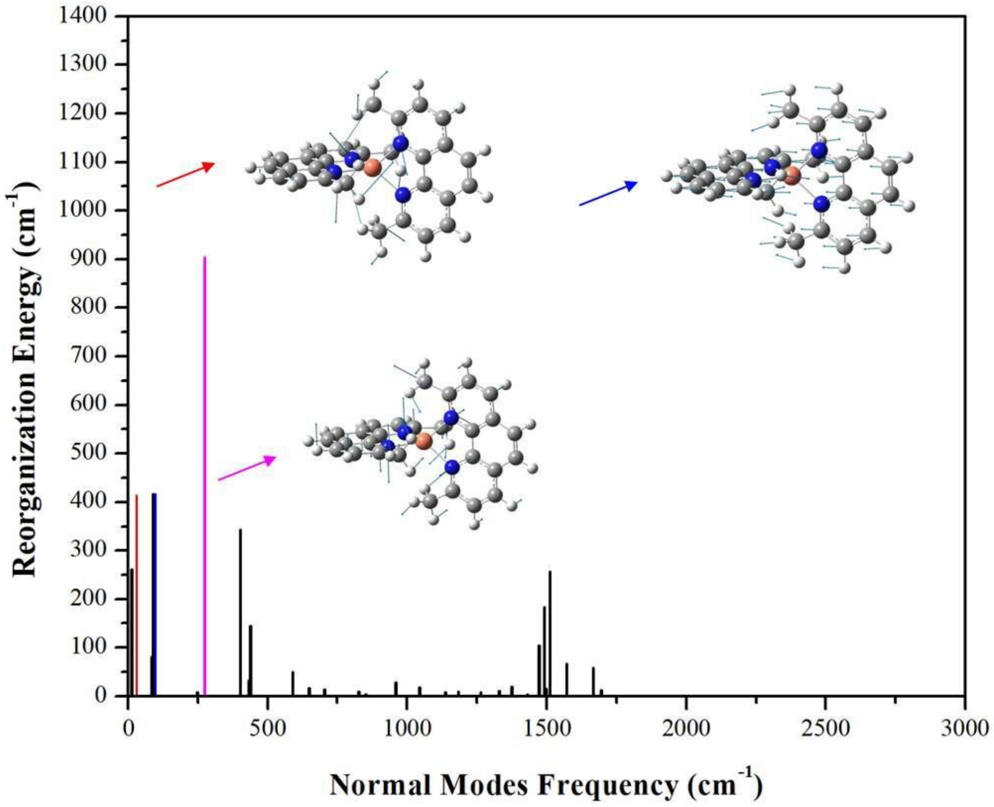

Figure S3. Diagrammatic illustration of the displacement vectors of the selected vibrational normal modes with the largest reorganization energies of complex 1.

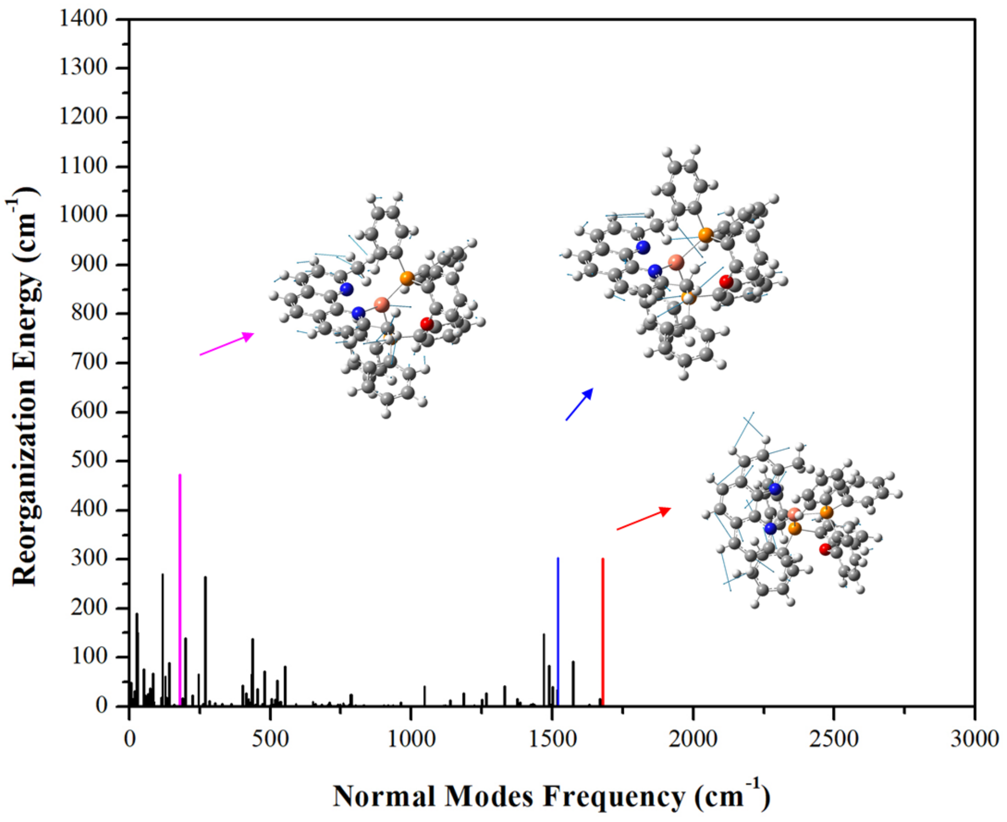

Figure S4. Diagrammatic illustration of the displacement vectors of the selected vibrational normal modes with the largest reorganization energies of complex 2.

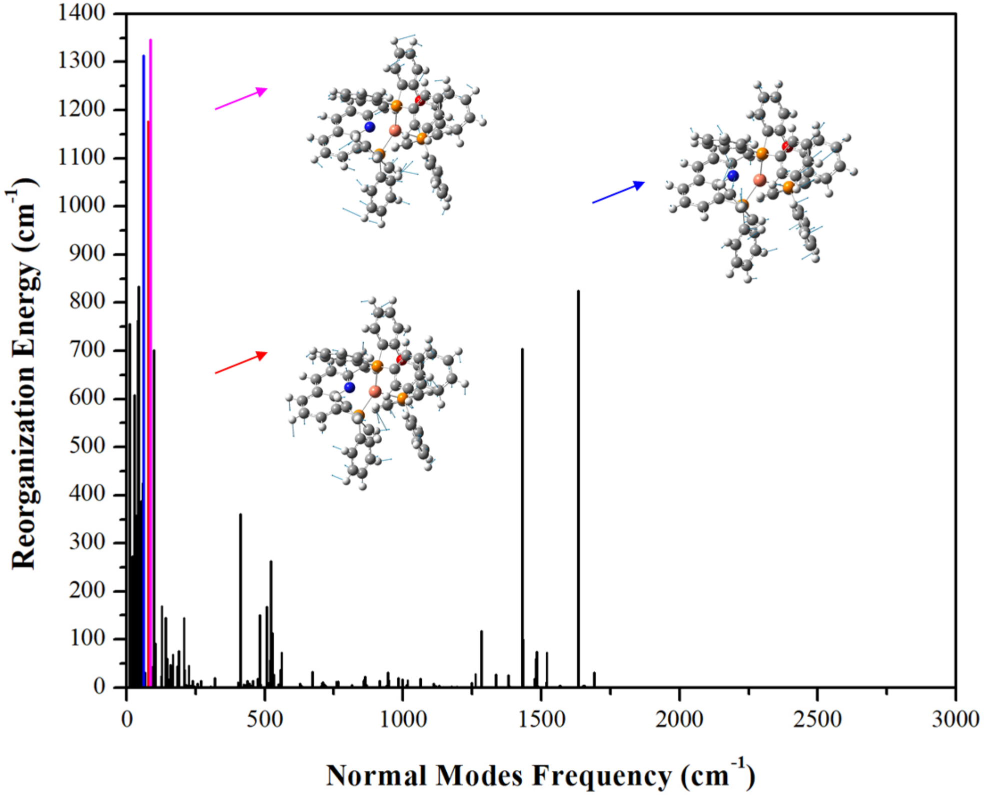

Figure S5. Diagrammatic illustration of the displacement vectors of the selected vibrational normal modes with the largest reorganization energies of complex **3**.

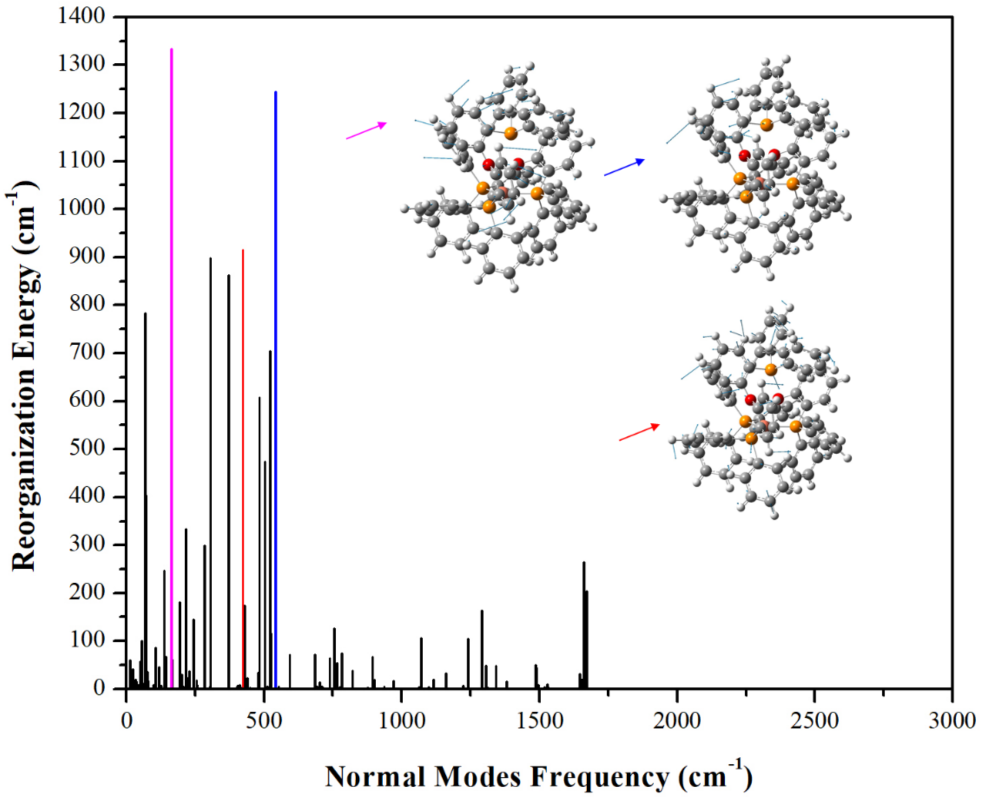

Figure S6. Diagrammatic illustration of the displacement vectors of the selected vibrational normal modes with the largest reorganization energies of complex **4**.
